# Factors That Determine Multiple Primary Cancers in the Adult Population in the United States

**DOI:** 10.7759/cureus.44993

**Published:** 2023-09-10

**Authors:** Francis Okeke, Valentine C Nriagu, Chisom M Nwaneki, Hezborn M Magacha, Nnamdi J Omenuko, Sandra Anazor

**Affiliations:** 1 Department of Epidemiology and Biostatistics, College of Public Health, East Tennessee State University, Johnson City, USA; 2 Department of Internal Medicine, Maimonides Medical Center, Brooklyn, USA; 3 Department of Internal Medicine, College of Public Health, East Tennessee State University, Johnson City, USA

**Keywords:** behavioral risk surveillance system, cancer treatment, smoking status, age at first cancer diagnosis, multiple primary cancers

## Abstract

Background: Cancer is a major public health problem worldwide and a leading cause of death in the United States. Multiple primary cancers mean that an individual has more than one cancer in the same or a different organ but does not include instances of metastasis of initial primary cancer. Several factors such as genetics, for example, *BRCA1* gene mutations, may predict multiple primary cancers. Factors such as the age at first cancer diagnosis may determine the outcome of multiple primary cancers. This study aims to determine factors that determine multiple primary cancers among the adult population in the United States.

Methods: This study uses data from the Behavioral Risk Factor Surveillance System 2021 dataset. The study included all individuals recently diagnosed with cancer (sample size = 9806). All age groups were included in this study. Measures included the outcome variable number of cancers and a major independent variable: age at first cancer diagnosis. Covariates included race, sex, smoking status, and cancer treatment. Descriptive, bivariate, and multivariate logistic regressions were conducted using a statistical analysis system. It was hypothesized that individuals with age at first diagnosis of cancer at a younger age have higher odds of having multiple primary cancers as compared to individuals diagnosed at an older age.

Results: The age group of 50-64 years had the highest percentage of only one cancer type (35.87%) and of two or more cancers (35.46%). A majority of females had two or more cancers (53.52%) as compared to males (47.48%). The majority of participants with only one cancer type (80.59%) and two or more cancers (88.61%) were of White non-Hispanic ethnicity. At the multivariate level, the age group under 18 years had 9.4% higher odds of having two or more cancers compared to the age group of 18-29 years (adjusted OR (AOR)=1.094, 95%CI=1.026-1.166; p-value=0.0057). The age group 65 years and above had 11.6% lower odds of having multiple primary cancers as compared to the age group of 18-29 years (AOR=0.884; 95%CI=0.859-0910; p-value=<0.0001). The Black non-Hispanic group had 73.8% lower odds of having multiple primary cancers as compared to White non-Hispanic respondents (AOR= 0.262; 95%CI = 0.228-0.301; p-value = <0.0001). Hispanic respondents had 59.8% lower odds of having two or more cancers as compared to the White non-Hispanic group (AOR= 0.402; 95%CI=0.390-0.413; p-value=<0.0001). Current smokers had 9.7% higher odds of having multiple cancers as compared to individuals who never smoked (AOR = 1.097; 95%CI=1.066-1.129; p-value=<0.0001). Former smokers had 24.2% higher odds of having multiple cancers as compared to individuals who never smoked (AOR=1.242; 95%CI=1.224-1.261; p-value=<0.0001). Individuals who were currently on treatment had 2.676 higher odds of having two or more cancers as compared to individuals not on treatment (AOR=2.676; 95%CI=2.629-2.724; p-value=<0.0001).

Conclusion: Multiple primary cancers have been on the increase recently following advancements in anticancer therapy and cancer screening and diagnosis technology. It is important that studies that aim to demonstrate risk factors and predictors of multiple primary cancers such as the age at first diagnosis, smoking status, and cancer treatment are encouraged among public health specialists.

## Introduction

Cancer is a major public health problem worldwide and the second leading cause of death in the United States [1]. In 2019, there were about 1.7 million cases of cancer reported and over 599,000 people died of cancer [2]. Cancer itself or its treatment causes immunosuppression, and this leads to cancer patients being more susceptible to infections compared to the general population. For example, cancer patients had a 3.5-fold increase in the risk of needing mechanical ventilation or ICU admission or dying compared with patients without cancer during the coronavirus disease 2019 (COVID-19) pandemic [3]. Over the years, efforts have been directed at and continue to be expended on identifying risk factors for cancer. Some cancers have been convincingly linked to specific environmental factors, e.g., smoking and lung cancer, human papillomavirus and cervical cancer, viral hepatitis, and hepatocellular carcinoma, etc. [4]. Nevertheless, some cancer patients develop more than one cancer type, which puts them at greater risk for cancer complications, more hospital visits, extended periods of treatment, and challenges in the choice of treatment. 

Multiple primary cancers 

Multiple primary cancers indicate that a patient or an individual has more than one cancer in the same or a different organ but do not include instances of metastasis of initial primary cancers. Tumors are considered multiple malignancies if they arise in different sites and/or are of a different histology or morphology group. This avoids misclassification of multifocal/multicentric tumors or metastases as multiple primaries [5]. Patients may have multiple cancers because of a mutation in a susceptible gene or because of exposure, for example, smoking and lung cancer [6]. Also, individuals who survive the first primary cancer may be susceptible to developing a second and, in rare cases, a third primary tumor due to a variety of factors, including cancer predisposition syndromes; e.g., Li Fraumeni syndrome, environmental exposures, and effects of treatment. Other reported risk factors for multiple primary tumors include Caucasian ancestry, young age at index cancer diagnosis, and positive family history [5]. Following the diagnosis of multiple cancers, specialists are left with the challenge of finding an anticancer therapy strategy that covers the cancers without increased risk of toxicity and relevant pharmacologic interactions and without a negative impact on the overall outcome [7]. 

Epidemiology of Multiple Primary Cancers

Improvements in detecting and diagnosing cancer at earlier stages and advances in treatment have yielded an increase in the population of individuals ever diagnosed with cancer, and with improved outcomes, there is an increasing population of multiple primary cancers [8]. Also, as the number of cancer survivors increases, the occurrence of multiple primary cancers is also likely to increase [9]. The incidence of multiple primaries in a cancer population varies between 2.4% and 8%, and up to 17% within 20 years of follow-up [5]. A study by Weir et al. 2013, demonstrated that during 1995-2008, multiple cancers (in both sexes) increased by 25.4%, and more multiple cancers were registered among males than in females [10]. Similarly, Qiao et al.'s study on multiple primary cancers in the United States, in 2020, showed that about 19.0% of cases overall were reported as multiple primary cancers, with a higher percentage seen in non-Hispanic White and older patients, and bladder melanoma having the highest percentage of cases reported as multiple primary cancer [11]. 

Age at Index Diagnosis

For cancer etiology in general, childhood cancers are thought to have a greater inherited predisposition, whereas environmental exposures accumulate with age and are likely to cause a greater proportion of adult cancers [12]. A leading cause of morbidity and mortality among individuals who have survived a first tumor is a second cancer, and thus, understanding risk factors and their causes is of clinical and public health importance [13]. The median age interval between the first and second cancer diagnosis was 17.8 years, with the shortest interval for subsequent leukemia at 8.9 years and the longest for subsequent small intestine and colorectal cancers at 23.1 years. A study by Inskip and Curtis, on new malignancies following childhood cancer in the United States (1973-2002), demonstrated that individuals who were diagnosed with cancer in childhood and survived had a six-fold increased risk overall of developing cancer compared with the general population [14]. Another study by Hayat et al., 2007, using the Surveillance Epidemiology and End Results (SEER) program data, demonstrated the age at index cancer diagnosis of adults who develop another cancer, with the lowest median age of 45 years for thyroid cancer and the highest median age of 71 years for colon and rectum cancer [8]. 

Objective of the study 

Previous studies have looked at primary cancers with few studies conducted on multiple primary cancers, which we can attribute to the low prevalence of multiple primary cancer in the past. Recently, the rate of multiple primary cancers has been on the increase with the advancement in anticancer therapy and the increased survival of cancer patients. Also, studies have looked at the effect of cancer treatment such as chemotherapy and radiotherapy causing multiple primary cancers, while the relationship between the age of first cancer diagnosis and multiple primary cancers has been poorly explored. This study aims to determine the prevalence of multiple primary cancers among adults in the United States and to determine the prevalence of multiple primary cancers among adults in the United States. Furthermore, this study will provide insights into the association between other predictors such as ethnicity, smoking status, and multiple primary cancers. 

## Materials and methods

The sample included individuals of all age groups that were currently diagnosed with cancer (sample size = 9806) using the 2021 Behavioral Risk Factor Surveillance System (BRFSS) Center for Disease Control and Prevention (CDC) dataset, which is a telephone survey that collects state data about United States residents regarding their health-related risk behaviors, chronic health conditions, and use of preventive services [15]. The target population represents 2.24% of the population. Individuals diagnosed with one cancer, two cancers, and three or more cancers were included in this study. All age groups were also included.

All variables used were recoded. First, we recoded the measure for the number of cancer types from one, two, and three or more cancers to only one cancer and two or more cancers. Responses such as don’t know, blanks, and refused were coded as missing. Secondly, the age at cancer diagnosis was coded as numeric data ranging from 1 to 97+ in BRFSS and this was recoded to different age groups which include: under 18 years, 18-29 years, 30-49 years, 50-64 years, and 65+years. The variable for sex was recoded similarly to that of BRFSS as male and female. The race variable was also re-coded in the same format as BRFSS. Additionally, for the smoking status measure, individuals who were current smokers and smoked every day and individuals who were current smokers but smoked sometimes in BRFSS were both coded as current smokers. Former smokers and never smoked on BRFSS were coded in a similar fashion in this study. Finally, for the last variable, cancer treatment, which asks specifically if respondents were currently receiving treatment, we coded the response Yes in a similar way as BRFSS, while responses such as "No, I have completed treatment", "No, I refused treatment", "No, I have not started treatment" and "Treatment was not necessary" were recoded as No. 

Statistical analysis

Descriptive, bivariate logistic, and multivariate logistic regression (using a backward stepwise method with criteria of inclusion set at P=0.2) were performed using SAS software Version 9.4 (2013; SAS Institute, Cary, North Carolina, United States). The sample weight _LLCPWT was used in all analyses. It was hypothesized that individuals with a younger age at first diagnosis of cancer have higher odds of having multiple primary cancers as compared to individuals diagnosed at an older age.

## Results

The characteristics of the study population by age at first diagnosis (Table 1) show the age group 50-64 years had the highest percentage of only one type of cancer (35.87%) and of two or more cancers (35.46%). The age group under 18 years had the lowest percentage of one type of cancer and two or more cancers (1.68% and 4.18%, respectively). Females had a higher percentage of two or more cancers (53.52%) as compared to males (47.48%). Additionally, the majority of the respondents with one cancer type (80.59%) and two or more cancers (88.61%) were of White non-Hispanic ethnicity. About 53.59% of respondents with only one cancer type never smoked and most individuals with two or more cancers (48.13%) never smoked. Finally, most respondents with only one cancer were not on treatment for cancer (86.76%), and 78.42% of individuals not on treatment for cancer had two or more cancers.

**Table 1:** Descriptive Analysis of Risk Factors and Multiple Primary Cancers

	Only one cancer	Two or more cancers
Variables	Frequency (%)	Frequency (%)
Age at First Diagnosis		
Under 18 years	82 (1.68%)	45 (4.18%)
18-29 years	385 (7.32%)	143 (8.04%)
30-49 years	1674 (23.52%)	525 (25.76%)
50-64 years	2789 (35.87%)	786 (35.46%)
65+	2723 (31.61%)	654 (26.56%)
Missing	428,887	
Gender		
Female	1511(55.27%)	424 (53.52%)
Male	1164 (44.79%)	380 (46.48%)
Missing	435,214	
Race		
White non-Hispanic	6225 (80.59%)	1884 (88.61%)
Black non-Hispanic	201 (4.13%)	25 (2.33%)
Hispanic	545 (11.23%)	88 (6.03%)
Multi non-Hispanic	177 (1.09%)	43 (0.89%)
Other non-Hispanic	356 (3.05%)	61 (2.15%)
Missing	429,088	
Smoking status		
Current smoker	628 (10.43%)	171 (11.26%)
Former smoker	2683 (35.98%)	848 (40.61%)
Never smoked	4292 (53.59%)	1125 (48.13%)
Missing	428,946	
Cancer Treatment		
Yes-on treatment	695 (13.24%)	343 (21.58%)
No-not on treatment	4834 (86.76%)	1242 (78.42%)
Missing	431,579	

Table 2 represents the bivariate and multivariate logistic regression analysis. 

**Table 2:** Bivariate and Multivariate Logistic Regression Determining Multiple Primary Cancers Note: In this table, "ref" refers to the Reference category, which serves as a point of comparison with other categories.

	Unadjusted (Bivariate)				Adjusted (Multivariate)		
Variables	Odds ratio	95% CI	p-value		Odds ratio	95% CI	p-value
Number of cancer types							
Only one cancer	ref	ref	ref		ref	ref	ref
Two or more cancers							
Age at first diagnosis			<0.0001				
Under 18 years	2.266	2.226-2.307			1.094	1.026, 1.166	0.0057
18-29 years	ref	ref			ref	ref	
30-49 years	0.997	0.986-1.008			1.205	1.172-1.239	<0.0001
50-64 years	0.900	0.890-0.909			1.137	1.106-1.168	<0.0001
65+ years	0.765	0.757-0.774			0.884	0.859-0.910	<0.0001
Gender			<0.0001				
Female	ref	ref			ref	ref	
Male	1.073	1.059-1.087			1.003	0.989-1.017	0.6859
Race			<0.0001				
White non-Hispanic	ref	ref			ref	ref	
Black non-Hispanic	0.513	0.504-0.522			0.262	0.228-0.301	<0.0001
Hispanic	0.488	0.483-0.494			0.402	0.390-0.413	<0.0001
Multi non-Hispanic	0.723	0.702-0.744			0.701	0.675-0.729	<0.0001
Other non-Hispanic	0.641	0.629-0.652			0.514	0.499-0.529	<0.0001
Smoking Status			<0.0001				
Current smoker	1.202	1.191-1.213			1.097	1.066-1.129	<0.0001
Former smoker	1.256	1.249-1.264			1.242	1.224-1.261	<0.0001
Never smoked	ref	ref			ref	ref	
Cancer treatment			<0.0001				
Yes, on treatment	1.803	1.788-1.819			2.676	2.629-2.724	<0.0001
No, not on treatment	ref	ref			ref	ref	

Age at first diagnosis

At the bivariate level, the age at diagnosis was significantly related to the number of cancers. At the multivariate level, controlling or adjusting for all other variables, the age group under 18 years had 9.4% higher odds of having two or more cancers (AOR = 1.094, 95%CI = 1.026-1.166; p-value = 0.0057) compared to the reference (ref) age group 18-29 years. The age group 30-49 years had 20.5% higher odds of having two or more cancers as compared to the age group 18-29 (AOR = 1.205; 95%CI= 1.172-1.239; p-value = <0.0001). Similarly, the age group 50-64 years had 13.7% higher odds of having multiple cancers as compared to the age group 18-29 years (AOR = 1.137; 95%CI= 1.106-1.168; p-value = <0.0001). The age group 65 and above had 11.6% lower odds of having multiple primary cancers as compared to the age group 18-29 years (AOR= 0.884; 95%CI = 0.859-0.910; p-value = <0.0001)

Gender

At the bivariate level, gender was significantly related to the number of cancers. At the multivariate level, controlling or adjusting for all other variables, the gender of respondents was not significantly related to the number of cancers (AOR = 1.003; 95%CI = 0.989-1.017; p-value = 0.6859)

Race

At the bivariate level, the race of respondents was significantly related to the number of cancers. Controlling or adjusting for all other variables, respondents of Black non-Hispanic ethnicity had 73.8% lower odds of having multiple primary cancers as compared to White non-Hispanic respondents (AOR= 0.262; 95%CI = 0.228-0.301; p-value = <0.0001). Also, Hispanic respondents had 59.8% lower odds of having two or more cancers as compared to the White non-Hispanic racial group (AOR= 0.402; 95%CI=0.390-0.413; p-value =<0.0001). The multiracial non-Hispanic group also had 29.9% lower odds of having two or more cancers as compared to the White non-Hispanic racial group (AOR=0.701; 95%CI=0.675-0.729: p-value = <0.0001). Other non-Hispanic racial groups had 48.6% lower odds of having two or more cancers compared to the White non-Hispanic group (AOR=0.514; 95%CI=0.499-0.529; p-value =<0.0001).

Smoking status

At the bivariate level, smoking status was significantly related to the number of cancers. Adjusting for other variables, current smokers had 9.7% higher odds of having two or more cancers as compared to individuals who never smoked (AOR = 1.097; 95%CI=1.066-1.129; p-value =<0.0001). Former smokers had 24.2% higher odds of having multiple (two or more) cancers as compared to individuals who never smoked (AOR=1.242; 95%CI=1.224-1.261; p-value=<0.0001).

Cancer treatment

At the bivariate level, cancer treatment was significantly related or associated with the number of cancers. Adjusting for other variables, individuals who were currently on treatment were 2.676 times as likely to have two or more cancers as compared to individuals not on treatment (AOR=2.676; 95%CI=2.629-2.724; p-value=<0.0001).

## Discussion

Our findings on the age at first diagnosis showed that individuals who were first diagnosed with cancer when under 18 years of age had 9.4% higher odds of having two or more cancers compared to the age group 18-29 years, and individuals who were first diagnosed with cancer at 65 years and above had 11.6% lower odds of having multiple primary cancers as compared to the age group 18-29. This finding is consistent with previous findings that show individuals diagnosed with cancer in childhood and survived, had a six-fold increased risk overall of developing cancer compared with the general population [14]. Similarly, our study is consistent with previous findings, such as Sigurdsson et al.'s study, which showed that the risk of thyroid cancer increased with radiation doses and both the increased and decreased risks were more pronounced in those diagnosed with a first primary malignant disease before 10 years of age than in those older than 10 years [16].

Our study showed that individuals who were currently on treatment were 2.676 times more likely to have two or more cancers as compared to individuals not on treatment. Similarly, Shimuzu et al. demonstrated new primary malignancies occurred in 36.9% of patients who survived esophageal squamous cell carcinoma after esophagectomy [17]. In a study by Tabuchi et al. in 2013, patients who smoked were seen to have a 59% and 102% higher risk for all second primary cancer and smoking-related second primary cancers, respectively, than patients who never smoked [18]. Cancer survivors who had recently stopped smoking had 18% and 26% less risk, respectively, for these second primary cancers than those who smoked at the diagnosis. This finding is consistent with our findings where individuals who were current or former smokers had 1.097 higher odds and 1.242 higher odds, respectively, of having multiple (two or more) cancers compared to those who never smoked. 

Strength of the study

The sample size of over 9000 participants which was weighted made our study statistically significant as a smaller sample size would have led to non-significant values. 

Limitations of the study

We could not control for the cancer types, type of treatment respondents received, and how long individuals had treatment. These are important cancer covariates that could help explain the factors that determine multiple primary cancers among adults in the United States. There were no measures that explained the treatment type and how long the treatment lasted. We, therefore, recommend research on treatment types and duration; this could help explain why cancer patients on treatment were more likely to develop multiple primary cancers as compared to cancer patients not on treatment. 

## Conclusions

Multiple primary cancers have been on the increase recently following advancements in anticancer therapy and cancer screening and diagnosis technology. Understanding the risk factors and genetic predispositions that contribute to the development of multiple primary cancers can help healthcare providers identify individuals who may be at higher risk for these types of cancers. For example, in our study, we noticed that younger individuals and cancer patients on cancer treatment were more likely to be diagnosed with multiple primary cancers. Patients with these risk factors need close follow-up and monitoring over the years. It is important that studies that aim to demonstrate risk factors, such as the age of diagnosis, smoking status, cancer treatment, type of treatment, duration of treatment, and effect on multiple primary cancers, are encouraged among public health specialists. This study will add to the literature and serve as a reference material for future studies.
